# MObile Technology for Improved Family Planning: update to randomised controlled trial protocol

**DOI:** 10.1186/1745-6215-15-440

**Published:** 2014-11-12

**Authors:** Chris Smith, Thoai D Ngo, Phil Edwards, Caroline Free

**Affiliations:** Department of Population Health, London School of Hygiene and Tropical Medicine, London, UK; Research, Monitoring and Evaluation Team, Marie Stopes International, London, UK

**Keywords:** Cambodia, contraception, family planning, mHealth, post-abortion family planning

## Abstract

**Background:**

This update outlines changes to the MObile Technology for Improved Family Planning study statistical analysis plan and plans for long-term follow-up. These changes result from obtaining additional funding and the decision to restrict the primary analysis to participants with available follow-up data. The changes were agreed prior to finalising the statistical analysis plan and sealing the dataset.

**Methods/design:**

The primary analysis will now be restricted to subjects with data on the primary outcome at 4-month follow-up. The extreme-case scenario, where all those lost to follow-up are counted as non-adherent, will be used in a sensitivity analysis. In addition to the secondary outcomes outlined in the protocol, we will assess the effect of the intervention on long-acting contraception (implant, intra-uterine device and permanent methods).

To assess the long-term effect of the intervention, we plan to conduct additional 12-month follow-up by telephone self-report for all the primary and secondary outcomes used at 4 months. All participants provided informed consent for this additional follow-up when recruited to the trial. Outcome measures and analysis at 12 months will be similar to those at the 4-month follow-up. The primary outcomes of the trial will be the use of an effective modern contraceptive method at 4 months and at 12 months post-abortion. Secondary outcomes will include long-acting contraception use, self-reported pregnancy, repeat abortion and contraception use over the 12-month post-abortion period.

**Discussion:**

Restricting the primary analysis to those with follow-up data is the standard approach for trial analysis and will facilitate comparison with other trials of interventions designed to increase contraception uptake or use. Undertaking 12-month trial follow-up will allow us to evaluate the long-term effect of the intervention.

**Trial registration:**

ClinicalTrials.gov NCT01823861.

## Update

### Background

This update outlines changes to the MObile Technology for Improved Family Planning (MOTIF) study statistical analysis plan and plans for long-term follow-up. These changes, subsequent to the publication of the protocol in *Trials* [1], result from discussions within the research team, recommendations from one author’s (CS) PhD examiners, and procurement of additional funding to conduct further follow-up. The changes were agreed and we informed *Trials* on 12 June 2014 prior to finalising the statistical analysis plan and sealing the dataset.

### Changes to 4-month analysis

The primary analysis will be restricted to participants with available follow-up data. This is a more common approach to trial analysis and is standard in trials of interventions designed to increase contraception use [2–4]. This change will therefore facilitate comparison of our results with other studies. The primary outcome remains the same: use of an effective modern method of contraception at 4 months post-abortion. For the primary analysis, we originally planned to consider all participants lost to follow-up as non-users of contraception. This is clearly an extreme-case scenario and is likely to underestimate contraceptive rates, as it is unlikely that all subjects lost to follow-up will be non-users. We now consider that this would be more appropriate for a sensitivity analysis. While the subset of subjects with follow-up data might not be representative of all subjects, comparison across arms should provide an internally valid comparison, providing follow-up rates are similar in the intervention and control arms.

In addition to the secondary outcomes outlined in the protocol, we will assess the effect of the intervention on long-acting contraception. Marie Stopes International Cambodia considers implant, intra-uterine device and permanent methods to be long-acting contraception. We anticipate that this additional secondary analysis will be of value to family planning service providers. The researchers conducting the data analysis will be blind to treatment allocation. A second independent researcher will check the analyses.

### Long-term trial follow-up

At recruitment, participants were given the option to consent for additional self-report follow-up of primary and secondary outcomes at 12 and 24 months, subject to the trial’s obtaining additional funding. All 500 trial participants provided consent for this potential additional follow-up. Subsequently CS obtained a Medical Research Council Population Scientist Fellowship, which included some funds for long-term MOTIF trial follow-up.

We obtained self-report follow-up data on 86.2% of participants at 4 months. Six participants withdrew from the study. This follow-up was conducted by two research assistants over a 5-month period.

To assess the long-term effect of the intervention, we plan to conduct 12-month trial follow-up on the remaining 492 trial participants, commencing July 2014. The follow-up questionnaire will be similar to that used at 4 months. We will collect information on current contraceptive use, repeat pregnancy or abortion, and contraception use over the 12-month post-abortion period. In addition, we will ask participants using contraception where they obtained it. We anticipate that it will take several months to conduct 12-month follow-up. Having achieved 86.2% follow-up at 4 months, we anticipate increased attrition at subsequent follow-up. Owing to limited resources, we will not complete follow-up at 24 months.

Outcome measures and analysis will be similar to those at the 4-month follow-up. The primary outcome at the 12-month follow-up will be use of an effective modern contraceptive method at 12-months post-abortion. This will be considered a second primary outcome, in addition to effective modern contraceptive use at 4 months. Secondary outcomes include long-acting contraception use, self-reported pregnancy, repeat abortion, and contraception use over the 12-month post-abortion period (to estimate the point prevalence of contraception use at any given time and of contraceptive discontinuation rates).

For the primary outcomes and for secondary outcomes with binary outcome measures we will estimate risk ratios with 95% confidence intervals and give a two-sided *P* value for statistical significance using the chi-squared test. We will perform Kaplan-Meier survival analysis to assess contraceptive discontinuation rates.

We will perform sub-group and sensitivity analysis as per the 4-month analysis. For sub-group analysis, we will assess whether the effect of the intervention varies according to age, urban versus rural residence, level of education and socioeconomic status. We will use the chi-squared test for heterogeneity at a 5% level of significance. If statistically significant overall heterogeneity is identified, relative risks and 99% confidence intervals will be estimated. Sensitivity analysis will include counting those lost to follow-up as non-users, per-protocol analysis, and analysis of clustering among participants from each clinic, as for the 4-month follow-up.

### Ethics

Ethical approval for this additional follow-up has been granted by the London School of Hygiene and Tropical Medicine ethics committee (reference number 6378–01), the Marie Stopes International ethics committee (reference number 002-13-Am14), and the Cambodia Human Research ethics committee (reference number 0193 NECHR).

## Conclusion

Restricting the primary analysis to those with follow-up data is the standard approach for trial analysis and will facilitate comparison with other trials of interventions designed to increase contraception uptake or use. Undertaking 12-month trial follow-up will allow us to evaluate the long-term effect of the intervention.

## Authors’ information

CS is a Clinical Research Fellow in the Department of Population Health at the London School of Hygiene and Tropical Medicine and a PhD candidate on the topic of mobile phone-based interventions to improve use of contraception.

TDN is Head of Research at Marie Stopes International.

PE is a Senior Lecturer in Statistics in the Department of Population Health at London School of Hygiene and Tropical Medicine.

CF is a Senior Lecturer in Epidemiology in the Department of Population Health at London School of Hygiene and Tropical Medicine.

## Abbreviations

MOTIF: MObile Technology for Improved Family Planning.
